# Time Course of Functional Recovery Following Single-Level Anterior Lumbar Interbody Fusion with and Without Posterior Instrumentation: A Retrospective Single-Institution Study

**DOI:** 10.3390/jcm14134397

**Published:** 2025-06-20

**Authors:** Tejas Subramanian, Stephane Owusu-Sarpong, Sophie Kush, Adin M. Ehrlich, Tomoyuki Asada, Eric R. Zhao, Kasra Araghi, Takashi Hirase, Austin C. Kaidi, Gregory S. Kazarian, Farah Musharbash, Luis Felipe Colón, Adrian T. H. Lui, Atahan Durbas, Olivia C. Tuma, Pratyush Shahi, Kyle W. Morse, Francis C. Lovecchio, Evan D. Sheha, James E. Dowdell, Han Jo Kim, Sheeraz A. Qureshi, Sravisht Iyer

**Affiliations:** 1 Department of Spine Surgery, Hospital for Special Surgery, 535 E 70th St, New York, NY 10021, USA; subramaniant@hss.edu (T.S.); owususarpongs@hss.edu (S.O.-S.); kushs@hss.edu (S.K.); ehrlicha@hss.edu (A.M.E.); asadat@hss.edu (T.A.); zhaoe@hss.edu (E.R.Z.); araghik@hss.edu (K.A.); hiraset@hss.edu (T.H.); kaidia@hss.edu (A.C.K.); kazariang@hss.edu (G.S.K.); musharbashf@hss.edu (F.M.); colonlui@hss.edu (L.F.C.); durbasa@hss.edu (A.D.); tumao@hss.edu (O.C.T.); shahip@hss.edu (P.S.); morsek@hss.edu (K.W.M.); lovecchiof@hss.edu (F.C.L.); shehae@hss.edu (E.D.S.); dowdellj@hss.edu (J.E.D.); kimh@hss.edu (H.J.K.); qureshis@hss.edu (S.A.Q.); 2 Weill Cornell Medical College, 1300 York Ave, New York, NY 10021, USA

**Keywords:** ALIF, arthrodesis, lumbosacral region, recovery of function, patient reported outcome measures, time factors, minimal clinically important difference

## Abstract

**Background/Objectives**: While anterior lumbar interbody fusion (ALIF) is a well-established treatment for degenerative lumbar spine pathology, the timing and pace of postoperative recovery remain poorly defined. Understanding these temporal trends is clinically important for setting patient expectations and optimizing postoperative care. **Methods**: This retrospective single-institution study evaluated functional recovery in patients undergoing primary, single-level stand-alone (SA) ALIF, or with percutaneous posterior instrumentation (PI). Patient-reported outcome measures (PROMs), including the Oswestry Disability Index (ODI), the Visual Analog Scale (VAS) for back and leg pain, and the SF-12 Physical Component Score (PCS), were assessed preoperatively and at 2 weeks, 6 weeks, 3 months, 6 months, 1 year, and 2 years postoperatively. Achievement of minimum clinically important difference (MCID), global rating change (GRC), and return-to-activity milestones were also analyzed. **Results**: A total of 143 patients were included (90 SA; 53 PI). PROMs showed significant improvement through 1 year. VAS-back improved by 2 weeks, while ODI and SF12 PCS initially worsened but improved after 6 weeks. By 6 months, over half of the cohort achieved MCID, with continued gains through 1 year. Most patients returned to driving and work, and over 90% discontinued narcotics. Recovery trajectories were comparable between groups, despite early delays in the instrumented cohort. **Conclusions**: These findings provide time-specific recovery benchmarks that can guide surgical decision-making, patient education, and expectations around functional milestones.

## 1. Introduction

The anterior lumbar interbody fusion (ALIF) approach is a widely used surgical technique for managing degenerative lumbar spine disease (i.e., disc degeneration, degenerative spondylolisthesis, foraminal stenosis). Its advantages include direct visualization of the disc space and a broad working corridor, which facilitate the placement of larger interbody cages—potentially improving segmental alignment and restoring disc height more effectively [1]. Numerous studies have reported favorable long-term outcomes and high patient satisfaction following ALIF [2,3]. The existing literature has largely focused on fusion rates, complication profiles, and long-term clinical improvement, often without quantifying when patients reach meaningful recovery milestones such as MCID or return to activity.

The trajectory of short- and mid-term functional recovery following ALIF remains poorly understood. The timing and pace of postoperative improvement are critical to patient satisfaction, return to activity, and management of expectations. Understanding how patients recover over time is critical for optimizing perioperative counseling and setting realistic expectations. Recent studies on other lumbar spine procedures, such as percutaneous transforaminal endoscopic discectomy and minimally invasive transforaminal lumbar interbody fusion (MIS-TLIF), have begun to map recovery patterns in finer detail, identifying early versus delayed improvements in pain and function [4,5]. However, given ALIF’s unique anterior approach, biomechanics, and instrumentation options may result in a distinct recovery profile, warranting dedicated investigation.

This study addresses a gap in the literature by providing a detailed analysis of postoperative recovery patterns over the first two years following single-level ALIF. The primary aim is to characterize trends in patient-reported outcome measures (PROMs) at multiple postoperative timepoints and to evaluate the proportion of patients achieving clinically meaningful improvement. Secondarily, we further aim compare outcomes between patients who underwent ALIF with versus without percutaneous posterior instrumentation. Our hypothesis is that regardless of construct type, patients will continue to experience meaningful improvement though 1 year, highlighting the time dependent recovery process following spine surgery. To our knowledge, this first study of its kind analyzing temporal trajectory of recovery following single-level ALIF. By providing granular data on recovery timing and functional milestones, this study offers clinically actionable benchmarks to inform surgical planning and patient counseling.

## 2. Methods

### 2.1. Study Design and Patient Inclusion

The retrospective analysis was conducted according to the guidelines of the Declaration of Helsinki and approved by the Institutional Review Board at the Hospital for Special Surgery (IRB# 2018–1599). A prospectively maintained institutional registry enrolls all patients undergoing degenerative or deformity spine surgery by one of twelve board-certified spine surgeons, following thorough informed consent to monitor postoperative recovery. From this surgical registry, patients who underwent primary single-level ALIF between 2017 and 2024 for the treatment of degenerative conditions of the lumbar spine were queried from. Both stand-alone ALIFs and anterior-posterior (A-P) ALIFs (ALIF followed by percutaneous posterior instrumentation) were included. The surgical technique of all ALIF procedures was standard as described in prior studies [6,7]. However, for the sake of cohort homogeneity, patients that required an additional posterior-based direct decompression were excluded. Patients with multilevel fusions and those with infection or oncological disease were also excluded. Patients had at least 1-year follow-up after surgery.

### 2.2. Data Collection

Data was collected and managed using REDCap (Research Electronic Data Capture, version 14.1.2) [8,9] hosted at Weill Cornell Medicine Clinical and Translational Science Center supported by the National Center For Advancing Translational Science of the National Institute of Health under award number: UL1 TR002384. The following data was collected and analyzed:

1. Demographic data: age, gender, body mass index (BMI), American Society of Anesthesiologists (ASA) class, age-adjusted Charlson Comorbidity Index (CCI)
2. Patient-reported outcome measures (PROMs): Oswestry Disability Index (ODI), Visual Analog Scale (VAS) back and leg, and 12-Item Short Form Survey Physical Component Score (SF-12 PCS) were collected at each follow-up time point (2 weeks, 6 weeks, 12 weeks, 6 months, and 1 year). Minimal clinically important difference (MCID) achievement was calculated for each PROM using thresholds of 12.8 ODI, 1.2 for VAS back, 1.6 for VAS leg, and 4.9 SF-12 PCS, as described by Copay et al. [10]
3. Global Rating Change (GRC): Patients were assessed at each follow-up time point using a single-question survey: “Compared to before your surgery, how do you feel?” with the following response options: (1) much better; (2) somewhat better; (3) the same; (4) somewhat worse; and (5) much worse. For analysis, responses of “much better” and “somewhat better” were categorized as “better,” while “somewhat worse” and “much worse” were classified as “worse”.
4. Operative and perioperative data: operative time, operated levels, estimated blood loss (EBL), length of stay (LOS)
5. Return to activities (RTA): Postoperative recovery was monitored by tracking patients’ return to daily activities. Those driving or working before surgery were asked at each follow-up visit whether they had resumed these activities, with tracking continuing until their return. Additionally, patients who required postoperative opioid analgesia were asked to report the date they discontinued use, if applicable.

### 2.3. Statistical Analysis

Descriptive statistics were presented as mean ± standard deviation for continuous variables and as count (percentage) for categorical variables. Changes in PROMs at each postoperative time point were assessed using paired-sample *t*-tests. Comparisons between stand-alone and A-P procedures were performed using independent-sample t-tests for continuous variables when normally distributed or Mann–Whitney U when non-normally distributed. Categorical variables were analyzed using the χ^2^ or Fisher’s exact test when the sample size was fewer than 10. Statistical significance was set at *p* < 0.05. All analyses were conducted using IBM SPSS Statistics version 25 (IBM Corp., Armonk, NY, USA).

### 2.4. Cohort Demographics

A total of 143 patients met the inclusion criteria. Among these, 90 (63%) patients underwent stand-alone ALIFs, and 53 (37%) patients underwent ALIFs with posterior instrumentation (Table 1). Patients in the A-P group were significantly older than those in the stand-alone group (58 vs. 50 years, *p* < 0.001). There was no significant difference in gender distribution (*p* = 0.812) or BMI (*p* = 0.272) between the groups. ASA class differed significantly between groups (*p* = 0.009), with a higher proportion of ASA 3 patients in the A-P group. The CCI was also significantly higher in the A-P group (2 vs. 1.2, *p* = 0.004). For surgical variables, EBL was not significantly different between the groups (*p* = 0.588). Conversely, operative time (248 vs. 103 min, *p* < 0.001) and LOS (76 vs. 47 h, *p* < 0.001) were significantly longer in the A-P group.

## 3. Results

**Figure 1:**
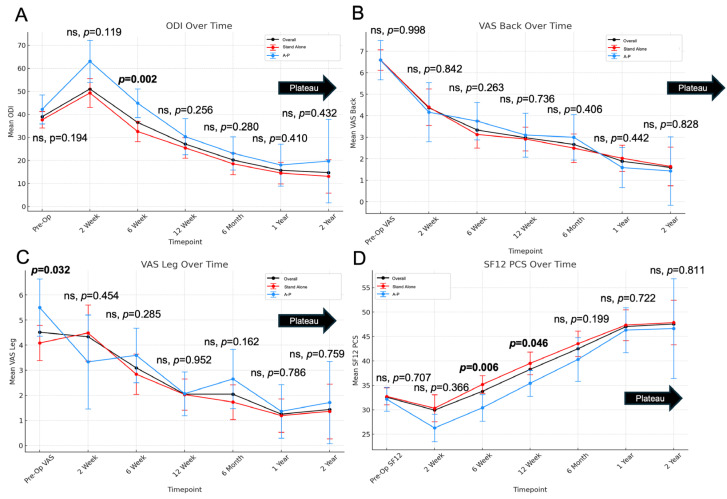
Temporal trends in (**A**) ODI, (**B**) VAS back, (**C**) VAS leg, and (**D**) SF-12 PCS after ALIF segmented by stand-alone and A-P groups, with *p*-values comparing stand-alone and A-P groups. ns denotes no statistical significance.

ODI worsened at 2 weeks postoperatively (39 to 51, *p* < 0.001) but improved by 6 weeks and plateaued at 1 year (Table 2, Figure 1A). At 6 weeks, A-P patients had greater disability than stand-alone patients (*p* = 0.002), with no differences at other time points. VAS-back improved by 2 weeks (6.6 to 4.4, *p* < 0.001) and continued improving through 2 years, with no group differences (Figure 1B). VAS-leg improved starting at 6 weeks (4.3 to 3.1, *p* < 0.001), plateaued by 1 year, and showed no between-group differences postoperatively, despite worse baseline scores in the A-P group (*p* = 0.032; Figure 1C). SF-12 PCS declined at 2 weeks (33 to 30, *p* = 0.05) but improved by 6 weeks and plateaued at 1 year (Figure 1D). Scores were higher in the A-P group at 6 and 12 weeks (*p* = 0.006, *p* = 0.046), with no differences thereafter.

**Figure 2:**
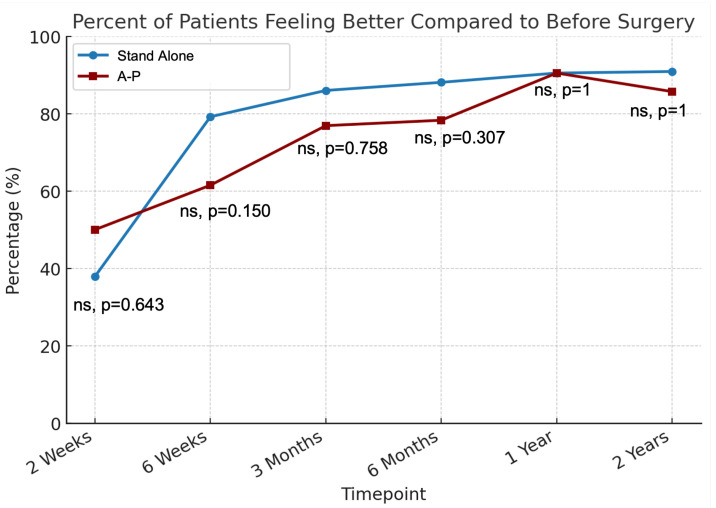
Percentage of patients feeling better compared to preoperative baseline at each postoperative timepoint. ns denotes no statistical significance.

At 2 weeks post-op, 39% of patients felt that they has improved, while 46% felt worse (Table 3, Figure 2). Improvement rates rose to 73% at 6 weeks, 83% at 3 months, and 85% at 6 months, peaking at 91% and 90% at 1 and 2 years, respectively. No significant differences were found between stand-alone and A-P groups at any time point.

**Figure 3:**
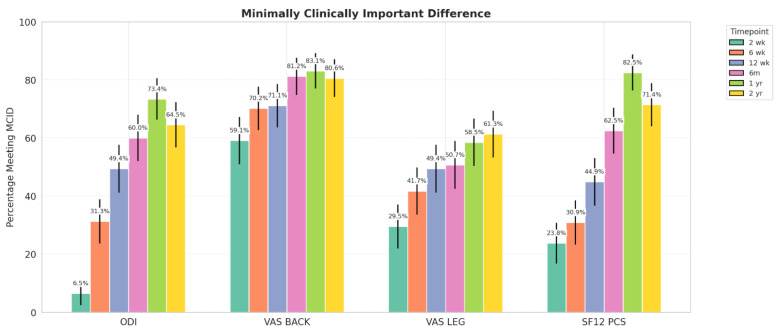
Percentage of patients achieving MCID for each PROM at each postoperative time point for the entire cohort. Error bars denote 95% confidence intervals calculated by the Wald approximation.

By 3 months post-op, nearly 50% of patients achieved MCID for each PROM (Figure 3, Table 4). VAS-back improved fastest, with >70% reaching MCID by 6 weeks, while ODI and SF-12 PCS lagged until 6 months. At 1 year, MCID achievement remained high across all PROMs, with sustained improvement at 2 years. No significant group differences were observed, except for higher VAS-leg MCID in the A-P group at 2 years (*p* = 0.026).

Postoperatively, 91% returned to driving, 86% to work, and 93% discontinued narcotics. Median time to driving, work, and narcotic cessation were 29, 27, and 12 days, respectively. There were no significant differences in recovery kinetics between the stand-alone and A-P groups (Table 5).

## 4. Discussion

In this retrospective study of patients undergoing single-level ALIF for degenerative lumbar spine pathology, we observed significant improvements in functional disability, back and leg pain, and physical health scores through the 1-year follow-up. Across all PROMs, including ODI, VAS-back, VAS-leg, and SF-12 PCS, patients demonstrated progressive improvement, with the majority achieving MCID and PASS by one year after surgery. These findings align with broader trends in lumbar fusion outcomes as shown in previous studies [5,11,12], reinforcing the idea that patient-reported recovery is a time-dependent process. By further exploring temporal trends in recovery after ALIF, our study aims to refine preoperative counseling and guide expectation management for patients before and after anterior lumbar interbody fusion surgery.

The time-dependent nature of recovery after lumbar fusion has significant implications for preoperative counseling. While early postoperative gains are encouraging, our study demonstrated that many patients may not achieve clinically meaningful improvement until well beyond 6 months postoperatively. The delay in reaching the plateau phase aligns with the findings of Shaikh et al., who reported that fewer than 50% of patients undergoing posterior lumbar fusion achieved the MCID for PROMIS scores by 6 months, despite continued improvement thereafter [13]. Similarly, Shahi et al. emphasized that both MCID and PASS are most often attained closer to the 1-year mark MIS-TLIF, reinforcing the gradual nature of patient-perceived recovery [5]. These data underscore the need for setting realistic recovery expectations during preoperative counseling, particularly for patients who may encounter slow or nonlinear symptom progression relief.

In our cohort, we observed rapid early improvements in pain scores. Patients significantly improved in their VAS-back scores at the 2-week follow-up, and almost 60% achieved MCID at that time point. VAS-leg scores improved substantially at the 6-week mark, with about 50% of patients achieving MCID for VAS-leg at this point. However, our findings for VAS pain scores do not parallel the recovery trends for the global disability PROMs in our cohort. For both ODI and SF-12 PCS, the average patient experienced significant worsening at the 2-week time point before beginning to improve at each subsequent follow-up. This early worsening of functional disability followed by later improvement is similar to the findings of Shahi et al. after MIS-TLIF [5]. No prior studies have specifically tried to answer the temporal trajectories of improvement following ALIF. Those that report postoperative PROMs usually do so within the context of comparing to alternative surgical interventions. This body of literature reports outcomes usually at 6 weeks or later with no focus on earlier recovery. For example, Nie et al. compared TLIF and ALIF in a worker’s compensation cohort [14]. In the ALIF cohort of only 34 patients, patients demonstrated significant improvements in physical function and back pain at the 6-week mark. Cha et al. studied the impact of preoperative symptom duration on ALIF outcomes, and while the authors did not conduct specific paired analysis, again, patients seemingly began improving, starting at the 6-week mark [15]. These studies have very different aims and therefore comparing them to the results of the present analysis is challenging.

The functional gains patients experienced during this period were reflected in real-world recovery milestones, with many resuming activities such as driving and working at a median of 4 weeks post-surgery. Recovery kinetics following spine surgery has become a focus of the recent literature [11,16], with most studies concentrating on posterior-based techniques. A recent systematic review of 46 patients’ recovery kinetics following ALIF found an average return to work time of 6 months [17]. Other studies have reported a quicker return to activities around the 3-month mark following ALIF [18,19]. While our data demonstrates even quicker recovery kinetics, these values are certainly individualized to patient comfort and can vary based on surgeon instructions. Regardless, it is important to counsel patients with this in mind to prevent discouragement related to early postsurgical disability.

Our subgroup stratification by surgical technique—stand-alone ALIF versus ALIF with percutaneous posterior instrumentation—revealed clinically meaningful differences in the recovery trajectory. Stratification between stand-alone and A-P ALIFs was based on surgeon/clinical discretion. This may introduce selection bias as surgeons may have preferentially selected to include posterior percutaneous instrumentation for patients with more advanced pathology or biomechanical instability, which could influence observed recovery trajectories. The purpose of the comparison was therefore not to report the superiority of one surgical technique versus the other regarding recovery but rather to provide meaningful recovery data for both. To that end, patients undergoing A-P ALIF had a significantly higher early postoperative disability (ODI and SF12-PCS), which may be attributed to the additional surgical burden associated with “more surgery” and a longer procedure [20,21]. Along the same lines, delayed disability recovery can result from increased soft tissue disruption, greater postoperative inflammation, or heightened nociceptive input from percutaneous screw placement may also contribute to early pain and delayed functional recovery. Prior studies have demonstrated elevated inflammatory markers and muscle trauma associated with posterior instrumentation, which may delay early mobilization and functional improvement [22,23]. Importantly, however, most of the other metrics, including long-term PROMs, return to activities, and global satisfaction, all followed similar trajectories of improvement. Potential transient differences in recovery kinetics should therefore not affect surgical decision-making concerning posterior instrumentation for ALIFs.

The clinical utility of these findings lies in the ability to inform both preoperative counseling and postoperative care. Patients frequently ask when they will feel better, be able to comfortably return to work, or discontinue narcotic pain medications [24,25]. While many experience meaningful pain relief within the first few weeks, our data show that functional recovery—as well as subjective improvement—often continues through 6 to 12 months. This underscores the importance of proactively aligning patient expectations with the time-dependent nature of recovery, especially during early follow-up when physical gains may precede perceived improvement. Moreover, the median return to work and driving in our cohort occurred well before the one-year mark, and most patients discontinued narcotic use by 2 weeks—concrete milestones that can reassure patients concerned about their recovery trajectory. Coupled with over 90% of patients reporting that they felt better than baseline at 1 year, these findings reinforce the durability of ALIF outcomes when surgical decision-making and patient selection are appropriately tailored. In the era of patient-centered care, future research should aim to identify patient-specific factors that influence recovery trajectories following ALIF and explore strategies to optimize these variables preoperatively to improve surgical outcomes and patient experience.

This study has several limitations. First, its retrospective design and single-center setting may limit generalizability. Retrospective analyses are inherently prone to selection bias and often reflect practice patterns unique to the institution or surgeon, potentially reducing external validity. Additionally, while PROMs offer valuable, patient-centered insight, they are vulnerable to ceiling effects and reporting bias, particularly in studies evaluating surgical recovery [26,27]. These limitations may functionally decrease the sensitivity of PROMs to detect subtle or incremental changes over time. Another limitation of this study is the absence of multivariate adjustment or propensity score matching to account for baseline differences between the stand-alone and instrumented cohorts, which limits our ability to draw causal inferences regarding the effect of surgical technique on recovery outcomes, a topic for future studies. Nonetheless, the goal of the present study was to provide general guidelines on postoperative improvement in patients after ALIF. These results may aid spine surgeons in providing some benchmarks for postoperative gains while maintaining the understanding that each patient is unique in pathology, treatment, and recovery.

## 5. Conclusions

This study characterized the recovery trajectory following single-level ALIF and compared outcomes between stand-alone and instrumented techniques. Patients experienced significant improvements in pain, function, and physical health through 1 year postoperatively, with early gains in VAS-back and more gradual improvements in VAS-leg, ODI, and PCS. Clinically meaningful recovery, as measured by MCID and PASS, was often achieved later in the postoperative course. Despite early functional delays in the instrumented group, overall recovery trajectories were comparable, supporting our hypothesis of meaningful improvement across both approaches. These findings offer practical benchmarks to inform patient counseling and perioperative planning.

## Figures and Tables

**Table 1:** Demographics and operative data.

Variable	Overall	Stand Alone	A-P	*p*-Value
n	143	90	53	
Age	52.78 ± 13.07	50.00 ± 12.17	57.51 ± 13.29	**<0.001**
Male	67 (46.9%)	40 (44.4%)	27 (50.9%)	0.812
BMI	27.70 ± 5.04	27.35 ± 4.90	28.31 ± 5.25	0.272
ASA Class				
1	15 (10.5%)	12 (75%)	3 (5.7%)	**0.009**
2	116 (81.1%)	75 (83.3%)	41 (77.4%)	
3	11 (7.7%)	2 (2.2%)	9 (17%)	
4	1 (0.7%)	1 (1.1%)	0 (0%)	
CCI	1.51 ± 1.59	1.22 ± 1.55	2 ± 1.56	**0.004**
EBL	118.87 ± 228.11	110.84 ± 275.18	132.36 ± 112.49	0.588
Operative Time	156.51 ± 80.73	103.27 ± 28.83	247.91 ± 54.88	**<0.001**
LOS (hours)	57.75 ± 37.93	47.26 ± 24.68	75.56 ± 48.71	**<0.001**
Surgical Level				0.273
L4-L5	13 (9.1%)	10 (11.1%)	3 (5.7%)	
L5-S1	130 (90.9%)	80 (88.9%)	50 (94.3%)	

**Table 2:** PROMs at preoperative and postoperative timepoints through two years for the full cohort.

	ODI	VAS-Back	VAS-Leg	SF12-PCS
Preoperative	39.02 ± 16.85	6.59 ± 2.34	4.49 ± 3.3	32.57 ± 7.36
2 Weeks	51.05 ± 20.11	4.37 ± 2.62	4.34 ± 3.47	29.93 ± 8.38
	**<0.001**	**<0.001**	0.345	**0.050**
6 Weeks	36.55 ± 18.53	3.34 ± 2.55	3.09 ± 3.22	33.78 ± 7.61
	**<0.001**	**<0.001**	**<0.001**	**0.001**
3 Months	27.12 ± 19.46	2.98 ± 2.41	2.04 ± 2.44	38.27 ± 8.77
	**<0.001**	0.843	**0.023**	**<0.001**
6 Months	20.23 ± 18.33	2.67 ± 2.51	2.05 ± 2.73	42.49 ± 9.88
	**<0.001**	0.058	0.692	**0.002**
1 Year	15.47 ± 18.75	1.88 ± 2.14	1.25 ± 2.37	47.02 ± 10.77
	**0.021**	**0.023**	**0.004**	**0.004**
2 Years	14.72 ± 20.37	1.59 ± 2.23	1.44 ± 2.64	47.57 ± 11.43
	0.135	**0.013**	0.608	0.817

**Table 3:** Percentage of patients feeling better, the same, or worse at each postoperative time point compared to the preoperative baseline.

	Overall			Stand Alone			A-P		
Timepoint	Better	Same	Worse	Better	Same	Worse	Better	Same	Worse
2 Weeks	39.4%	15.2%	45.5%	37.9%	13.8%	48.3%	50.0%	25.0%	25.0%
6 Weeks	73.4%	12.7%	13.9%	79.2%	13.2%	7.5%	61.5%	11.5%	26.9%
3 Months	82.9%	6.6%	10.5%	86.0%	6.0%	8.0%	76.9%	7.7%	15.4%
6 Months	84.6%	3.1%	12.3%	88.1%	2.4%	9.5%	78.3%	4.3%	17.4%
1 Year	90.5%	1.6%	7.9%	90.5%	0.0%	9.5%	90.5%	4.8%	4.8%
2 Years	89.7%	3.4%	6.9%	90.9%	0.0%	9.1%	85.7%	14.3%	0.0%

**Table 4:** The percentage of patients achieving MCID for each PROM at each postoperative time point is segmented by stand-alone and A-P groups. * *p* = 0.026.

	Stand Alone				A-P			
	ODI	VAS-Back	VAS-Leg	SF-12 PCS	ODI	VAS-Back	VAS-Leg	SF-12 PCS
2 Weeks	7.1%	57.5%	25.0%	26.3%	0.0%	75.0%	75.0%	0.0%
6 Weeks	31.0%	70.7%	41.4%	33.3%	32.0%	69.2%	42.3%	25.0%
12 Weeks	46.6%	75.4%	43.9%	48.1%	56.0%	61.5%	61.5%	37.5%
6 Months	60.4%	85.1%	48.9%	65.1%	59.1%	72.7%	54.5%	57.1%
1 Year	69.8%	81.8%	54.5%	78.6%	81.0%	85.7%	66.7%	90.5%
2 Years	62.5%	79.2%	50% *	71.4%	71.4%	85.7%	100% *	71.4%

**Table 5:** Return to driving/work and discontinuation of narcotics after surgery. Days required is reported as median [interquartile range].

**Variable**	**Overall**	**Stand Alone**	**A-P**	***p*-Value**
Driving	n = 81	n = 58	n = 23	
Return to Driving	91.36%	93.10%	86.96%	0.375
Days Required	29 [14–48.5]	28 [12–46]	39 [24–62]	0.080
Working	n = 70	n = 51	n = 19	
Return to Working	85.71%	88.24%	78.95%	0.323
Days Required	29 [12–61]	27 [12–57.5]	35.5 [13–91]	0.267
Opioids	n = 88	n = 62	n = 26	
Discontinuation of Narcotics	93.18%	93.55%	92.31%	0.903
Days Required	12 [4.5–40]	9 [3–21]	22 [7–60]	0.055

## Data Availability

The data presented in this study are available on request from the corresponding author due to institutional policies and patient confidentiality regulations. Data sharing is restricted to protect sensitive patient information in compliance with ethical guidelines and IRB-approved protocols.
